# Challenges associated with follow-up care after implementation of an HPV screen-and-treat program with ablative therapy for cervical cancer prevention in Iquitos, Peru: a mixed methods study

**DOI:** 10.21203/rs.3.rs-3210614/v1

**Published:** 2023-08-23

**Authors:** Rachel Morse, Joanna Brown, E. Jennifer Ríos López, Bryn A. Prieto, Anna Kohler-Smith, Karina Gonzales Díaz, Magaly Figueredo Escudero, Daniel Lenin del Cuadro, Giannina Vásquez del Aguila, Henrry Daza Grandez, Graciela Meza, J. Kathleen Tracy, Patti E. Gravitt, Valerie A. Paz‐Soldan, The Proyecto Precancer Study Group

**Affiliations:** Department of Tropical Medicine, Tulane University School of Public Health and Tropical Medicine; Asociación Bené ca PRISMA; Asociación Bené ca PRISMA; Department of Tropical Medicine, Tulane University School of Public Health and Tropical Medicine; Asociación Bené ca PRISMA; Department of Cancer Control and Prevention, Gerencia Regional de Salud de Loreto; Department of Cancer Control and Prevention, Gerencia Regional de Salud de Loreto; Department of Cancer Control and Prevention, Gerencia Regional de Salud de Loreto; Department of Cancer Control and Prevention, Gerencia Regional de Salud de Loreto; O cina de Servicios de Salud, Gerencia Regional de Salud; Facultad de Medicina Humana, Universidad Nacional de la Amazonía Peruana; Department of Medicine, University of Vermont College of Medicine; Department of Epidemiology and Public Health, University of Maryland School of Medicine; Department of Tropical Medicine, Tulane University School of Public Health and Tropical Medicine

**Keywords:** Lost to follow-up, Cervical Cancer, HPV Screening, Screen-and-treat

## Abstract

**Background:**

Cervical cancer is a preventable cancer; however, decreasing its prevalence requires early detection and treatment strategies that reduce rates of loss to follow-up. This study explores factors associated with loss to follow-up among HPV-positive women after implementation of a screen-and-treat approach with visual triage and ablative therapy for cervical cancer prevention in Iquitos, Peru.

**Methods:**

We conducted semi-structured interviews with nurse-midwives (n = 15) working in cervical cancer prevention and women (n = 24) who were recorded as lost to follow-up after positive HPV results. We used the Health Care Access Barriers Model to guide analysis. We utilize manifest content analysis to describe barriers to follow-up according to the nurse-midwives and thematic analysis to report themes from the women’s perspectives. We also report the steps and time taken to contact women and report discrepancies and concordances between nurse-midwives and women regarding reasons for loss to follow-up.

**Results:**

Women in this study expressed a desire to receive treatment. Barriers, including fragmented and incomplete registry systems, made receiving follow-up care more challenging. Nurse-midwives faced structural barriers in attempting to deliver positive results to women who were challenging to contact, and women did not have clear knowledge of how to receive their HPV results. Women faced cognitive barriers including a lack of understanding about HPV results and treatment procedures, fear or anxiety about HPV or treatment, and confusion about the follow-up process. Women also reported having important work matters as a barrier. Reported financial barriers were minimal. There was agreement between women’s and nurse-midwives’ reported barriers to follow-up in slightly over half of the cases.

**Conclusion:**

This study highlights the barriers to follow-up after implementation of a primary-level HPV-based screen-and-treat approach. While some barriers that have previously been associated with loss to follow-up were not observed in this study (e.g., financial), we emphasize the need for screen-and-treat programs to focus on strategies that can address incomplete registry systems, structural challenges in results delivery, cognitive barriers in understanding results and treatment, and work-related barriers.

## Background

Cervical cancer is the second most common cancer among women in South America (1). In the Loreto district of Peru, cervical cancer is the primary contributor to cancer-related deaths among women, and the mortality rate from cervical cancer in this region is the second highest in Peru at approximately 14.9 per 100,000 (1–3). However, cervical cancer can be effectively prevented by utilizing vaccines for human papillomavirus (HPV) - the main cause of cervical cancer - and early detection and treatment (EDT) programs (4, 5). Successful implementation of vaccination and EDT programs in Peru requires adaptation to the complexities of the healthcare system. This adaptation is needed to ensure access to effective screening, timely follow-up for abnormal screening results, and prompt treatment for those requiring it.

In 1998, the Peruvian Ministry of Health declared cervical cancer prevention a national priority and planned to implement freely available HPV vaccination and EDT programs (6). Despite this prioritization, cervical cancer rates in Peru have not declined (3). One factor contributing to this stagnancy is the number of screen-positive women who are lost to follow-up (LTFU). Women who are LTFU do not reach an appropriate conclusion in their continuum of care by either receiving treatment or a negative confirmatory screening test (3, 7).

To facilitate strengthening of the cervical cancer EDT program in the Loreto district of Peru, an implementation science project, Proyecto Precancer, worked with local health authorities to create a new EDT approach: a screen-and-treat program, based at the primary-level, with HPV testing followed by visual triage and ablative therapy for eligible women. If women are ineligible for ablative therapy, they are referred for specialist hospital-level follow-up (see Morse et al. (8) for further details). Prior to implementation of this screen-and-treat approach, Proyecto Precancer’s monitoring and evaluation data examined the LTFU rate among women who tested positive following visual inspection with acetic acid (VIA) in the Micro Red Iquitos-Sur (MRIS) health network of Loreto. Between January 2018 and June 2019, 69.8% (120/172) of these women were LTFU (9).

In the MRIS, women who were interviewed to help understand this high rate of LTFU described a strong desire to complete the continuum of care but encountered a fragmented, burdensome system that continuously impeded their care. They faced cognitive barriers such as lack of knowledge about cervical cancer, misunderstandings about screening results or treatment, lack of awareness of the follow-up process, unclear communication from staff, and preconceived notions about the challenges at the hospital-level. They also encountered structural barriers including challenges receiving results or scheduling appointments, unavailability of providers, long wait times, complicated care processes, and broken equipment, and financial barriers including out-of-pocket payments and costs related to travel or missing days of work (10). These hospital-level barriers are also commonly found in other low- and middle-income settings (11–18). Barriers to care in a primary-level HPV based screen-and-treat program were studied in Kenya from the perspective of healthcare providers (19, 20), which identified cognitive barriers among women including a lack of knowledge about HPV and cervical cancer, structural barriers such as a lack of supplies and lack of adequate staffing, and financial barriers including the cost of transport to health facilities.

Proyecto Precancer’s implementation of the screen-and-treat approach aimed to address many of the reasons women are LTFU at hospital-level by task shifting the management of cervical cancer from the hospital-level to the primary-level facilities. Following implementation of this program in 2019, our monitoring and evaluation data between July 2019 and February 2020 revealed a LTFU rate of 30.0% (174/580) among women with a positive HPV result in the MRIS (9). While this LTFU rate is a considerable improvement over the LTFU rate of 69.8% before implementation, women are still being LTFU in the screen-and-treat approach.

This study aimed to explore the factors associated with LTFU in a screen-and-treat approach with visual triage and ablative therapy at the primary-level from the perspectives of women who are recorded as LTFU and nurse-midwives working in cervical cancer prevention in Iquitos, Peru. This study incorporates the perspectives of multiple stakeholders and provides insight into changes that can further reduce rates of LTFU in screen-and-treat programs at the primary-level.

## Methods

To ensure we obtained a detailed understanding of LTFU in the system, we conducted semi-structured interviews with nurse-midwives working in cervical cancer care and women who were recorded as LTFU in the MRIS on the same topic: reasons for LTFU.

### Setting:

This study was conducted in an Amazonian region of Peru, specifically in the Micro-Red Iquitos Sur (MRIS) health network (population 127,000) in Iquitos (population 400,000). Iquitos is the capital city of the Loreto district; it is the largest city in the world that can only be reached by plane or by boat – there are no roads to the city. Fishing, agriculture, logging, oil extraction, tourism, and small businesses are the main sources of income in Iquitos.

The MRIS is home to 20,000 women between 30–49 years who are eligible for the screen-and-treat approach using HPV testing and ablative therapy (21). The public health facilities in the MRIS are covered by the Seguro Integral de Salud (SIS) [Comprehensive Health Insurance]. SIS is a public healthcare insurance program that provides full or partially subsidized insurance to people in Peru living in poverty or extreme poverty. In Loreto, 67% of the population has SIS coverage (22).

Within the MRIS, there are 17 health facilities ranging in size and capacity. Some larger centers are staffed with doctors and nurse-midwives and have laboratories while other smaller centers are staffed only by one nurse-midwife without laboratories. The nurse-midwives (or ‘obstetras’) provide women’s health services including cervical cancer screening. Women are able to access care at their local health facility and, if needed, can be referred to a larger health facility or one of two regional hospitals.

### Participant selection, procedures, and data collection:

**Figure 1:**
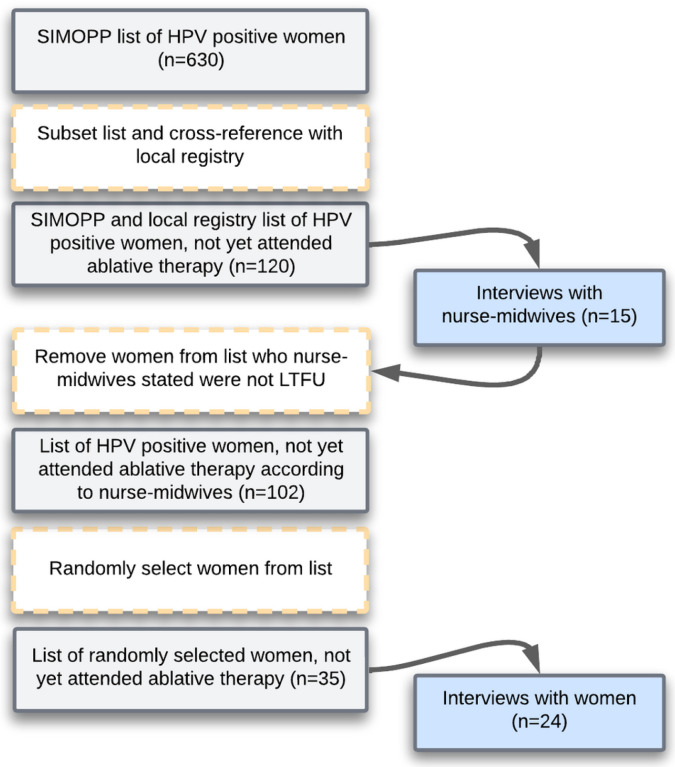
A summary of the processes for data collection. The yellow boxes depict action taken during the data collection process, the gray boxes describe the data source, and the blue boxes show the data collection.

We used a purposive sampling process (summarized in Fig. 1). We began by generating a list of 630 women who had a positive HPV result between May 2019 and November 2020 recorded in SIMOPP, a Proyecto Precancer monitoring system. We then subset this list to include only women who had no recorded evidence of treatment within 10 months of their positive HPV result. We also cross referenced this list with nurse-midwives’ handwritten notebooks on treatment attendance and created a final list of 120 women who had not attended treatment.

We then invited nurse-midwives from 16 of the MRIS health facilities to complete semi-structured interviews regarding their perspectives on why the 120 women were LTFU. One health facility was excluded as they had no women who were LTFU. One nurse-midwife worked at two health facilities, and as a result, we interviewed 15 nurse-midwives between July 2021 and August 2021. All nurse-midwives provided informed consent prior to interviews with one of two Peruvian researchers (J.B., E.J.R.L.). Interviews were completed in Spanish over the phone or in person in a private area of the health center. The nurse-midwives were asked – to the best of their knowledge – about their attempts to contact patients, whether treatment had been scheduled, and the patient’s desire or lack thereof to attend treatment. The researchers documented responses using a semi-structured form.

After the interviews, it became apparent that 18 women who were previously recorded as LTFU had attended triage and either received ablative therapy or were referred to the hospital and received hospital-level treatment, despite there being no record of this. We then removed these women from the list of 120 women who were LTFU and generated a new list with 102 women who were still recorded as LTFU. We then randomly selected 35 of these women to participate in semi-structured interviews. We attempted to contact women over the phone (if they owned one) or by a house visit to coordinate interviews. However, we were unable to contact 11 women for reasons discussed in the results. The remaining 24 women agreed to take part in an interview.

The women’s interviews were conducted in Spanish between August 2021 and February 2022 over the phone or in a private location in the participants’ homes, after they provided informed consent. The interviews focused on women’s understandings of and experiences with HPV, HPV screening, women’s desire to receive care, and women’s emotions about and experiences with the care process. If the woman being interviewed had not yet received her positive HPV result, the interviewer (E.J.R.L.) explained that the HPV test was positive, provided counseling, explained that the woman could attend treatment, if she would like, and provided help scheduling treatment, if requested. In the case where a woman had received their positive HPV result but did not know about available treatment, the interviewer described the treatment and provided help scheduling an appointment, if requested. All interviews were audio recorded and transcribed verbatim. The interviewer also took field notes.

### Data analysis:

We used the Health Care Access Barriers (HCAB) Model to guide qualitative and quantitative analysis. The HCAB is a framework developed to classify, analyze, and report measurable and modifiable health determinants categorized into three types of barriers: financial, structural, and cognitive (23).

During the quantitative analysis, the researchers (R.M.M, J.B.) used manifest content analysis to categorize the women discussed in the nurse-midwife interviews into groups according to the barrier stated by the nurse-midwife that resulted in their LTFU, if this barrier was known. Each of the barriers was then categorized according to the HCAB, if applicable or was categorized as other, if not applicable.

To consider challenges in contacting women, we report the steps and time taken to contact women and report discrepancies and concordances between nurse-midwives and the interviewer. We note whether the nurse-midwives and the interviewer were able to contact the same women.

In Dedoose Version 8.0.35, the researchers (R.M.M, J.B.) analyzed the interviews using thematic analysis and developed a codebook using the HCAB. The codebook was adjusted as interview transcripts were reviewed. Ten transcripts were double coded, and any coding differences were discussed between the coders and resolved by consensus. Once all transcripts were coded, the coders reviewed the transcripts to ensure the coding was consistent with the final codebook.

Finally, to examine discrepancies and concordances between nurse-midwives and women, we compared findings from these two groups; specifically, we report whether the nurse-midwives’ reasons stated for why each woman was LTFU matched what each woman stated as her reason why she was LTFU.

## Results

### Nurse-midwife interviews:

**Figure 2:**
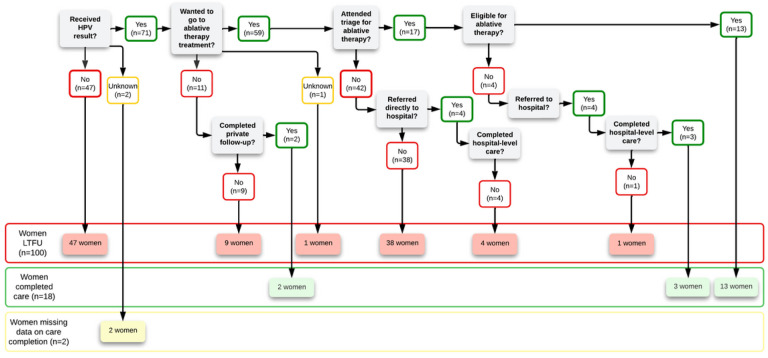
Continuum of care model depicting where women were LTFU or completed care according to the nurse-midwives

Out of 120 women who were LTFU, two of the women had missing information regarding completion of follow-up care following the nurse-midwife interviews. Of the remaining 118 women, nurse-midwives reported that 18 women reached an endpoint of care despite there previously being no record of reaching an endpoint following their positive HPV test: 13 received ablative therapy, three received hospital-level treatment, and two received a negative confirmatory screening test through private follow-up care. Figure 2 summarizes where in the continuum of care each woman was LTFU (n = 100) or completed care (n = 18) according to the nurse-midwives. Finally, of these 100 women reported by the nurse-midwives as LTFU, one attended triage for ablative therapy and was referred to the hospital, and four were referred directly to the hospital. These five women were LTFU at the hospital-level, and we focus on the 95 women LTFU at the primary-level below.

**Figure 3:**
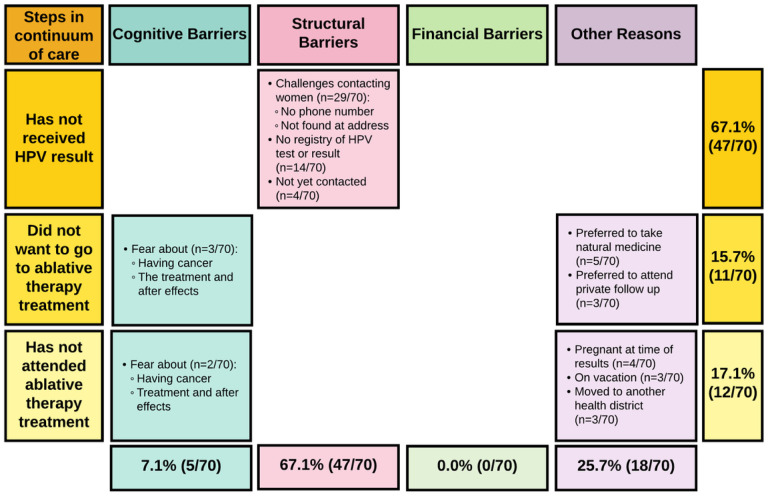
Summary of barriers to the completion of care according to the nurse-midwives

Of the 95 women who were LTFU at the primary-level, the nurse-midwives provided a reason for why the woman was LTFU in 70 cases; the reasons were unknown to the nurse-midwife for the other 25. According to the nurse-midwives, 47 of the 70 women were LTFU due to three main structural barriers: challenges in contacting the women, a lack of registry of the HPV test results at the primary-level (e.g., a new nurse-midwife without access to the former nurse-midwife’s notebook), or pending results delivery for women who had not yet been contacted. Eighteen of the 70 women were LTFU due to other reasons (e.g., vacation, being pregnant at the time of result delivery, preference for natural medicine). Five of the 70 women were LTFU due to two main cognitive barriers: fear of cancer or of treatment and aftereffects. No women were LTFU due to financial barriers (Fig. 3).

### Contacting women:

**Figure 4:**
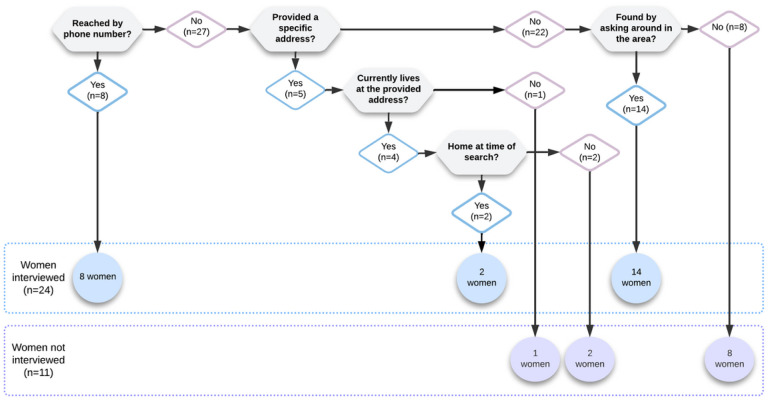
Summary of the process of finding women for interviews

Of the 35 women who were selected to take part in interviews, we were only able to contact 24, reflecting an important challenge in the system. It took the interviewer an average of 3.1 hours and an average of 2.2 attempts searching in-person to contact each of the 24 women, find where she lived and arrive at the address (if the interview was conducted at the woman’s home). Further details about the process of finding women for interviews are described in Fig. 4.

**Figure 5:**
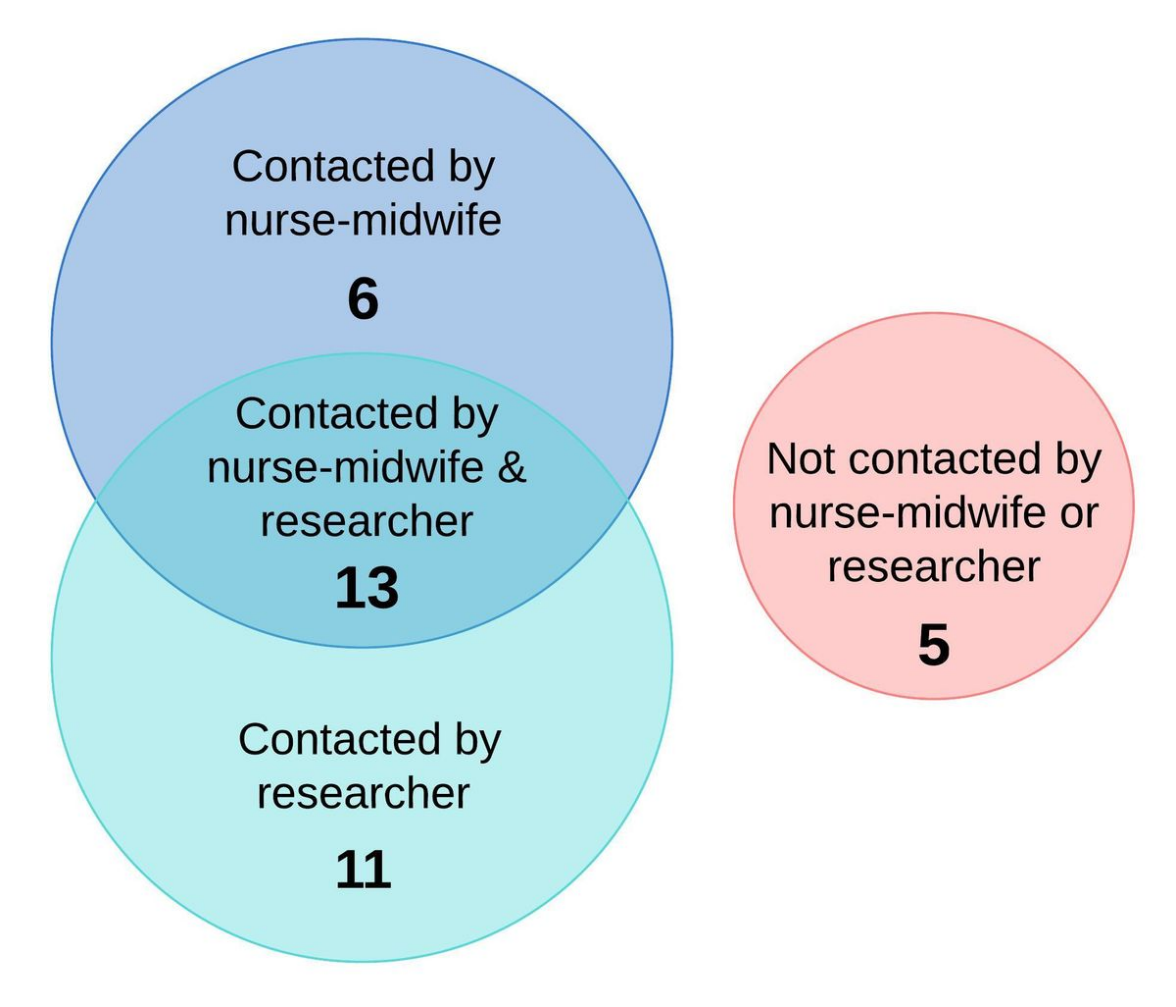
Summary of the number of women successfully contacted by nurse-midwives, the researcher, both, or neither

The nurse-midwives and the researcher were both able to contact 13 (out of 35) of the randomly selected women (Fig. 5): six were only able to be contacted by the nurse-midwives, 11 were only able to be contacted by the researcher, and 5 were unable to be contacted by the nurse-midwives nor the researcher.

### Interviews with Women:

#### Sample characteristics:

We interviewed 24 women (age_mean_ 39.6 years) identified as LTFU. Fifteen (62.5%) women were from urban health centers, six (25.0%) from peri-urban health centers, and three (12.5%) from rural health centers. Out of the 22 women who reported where their HPV test was done, seven (31.8%) had their test done in the community (e.g., during a campaign where nurse-midwives went door-to-door) and 15 (68.2%) had their HPV test done at the health center. Thirteen (54.2%) women had not received their HPV result. Of those who had received their HPV result (n = 11), seven (63.6%) received it at the facility, two (18.2%) over the phone, and two (18.2%) during a house visit by a nurse-midwife.

Five of the 24 women reported that despite there being no record of reaching an endpoint of care, they did reach an endpoint; two women received hospital-level treatment and three women received ablative therapy. Two of the women who completed care received at least part of their follow-up in a private facility:

Well, when I had the molecular test done, parallel to that, I had a biopsy done privately. With that biopsy, plus the molecular test, it was evident that I had cancer; so, I was referred to the Regional Hospital.

(Participant 13, completed treatment)

**Figure 6:**
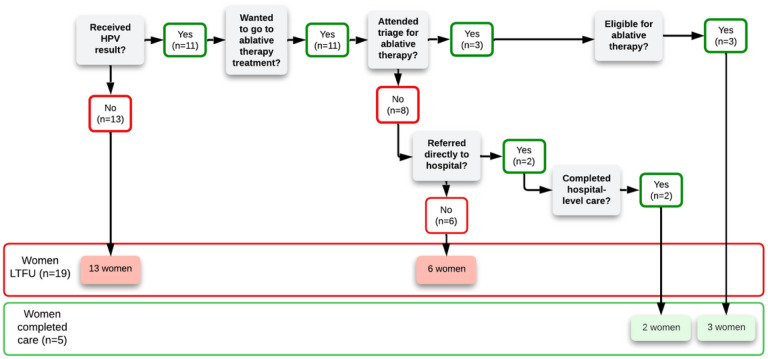
Continuum of care model depicting where women were LTFU or completed care according to the women themselves

The five women who completed the continuum of care are not excluded from the following discussion as they spoke about important barriers to follow-up. Figure 6 summarizes where in the continuum of care each woman was LTFU or completed care according to the women (whereas Fig. 2 is from the nurse-midwives’ perspectives).

#### Main barriers to completing care:

All 19 (out of 19) women who were LTFU expressed a desire to receive treatment. One woman described this as: *“I am positive for this disease [HPV], but I would like to be cured,” (Participant 18, LTFU). Another stated, “Well, it motivates me a lot because as women, we can’t have this disease… It’s better to go to our health center and have the doctor’s treatment,”* (Participant 7, LTFU). However, despite showing a strong desire to receive treatment, the women were faced with cognitive, structural, financial, and other barriers throughout the continuum of care.

#### Cognitive:

Five main cognitive barriers emerged: lack of understanding about the HPV result, fear or anxiety about HPV, lack of awareness of or confusion about the follow-up process, lack of understanding of treatment procedures, and fear or anxiety about treatment.

Nine women showed a lack of understanding of their HPV result. For example, one woman stated after she received her HPV result, *“The lady told me that I had infections only,”* (Participant 9, LTFU). Another woman expressed confusion about the meaning of the result by stating that she was told her HPV result was negative: *“She [the nurse-midwife] told me, ‘I don’t think it came back positive, it came back good,’”* (Participant 14, LTFU).

In some of these cases, the lack of understanding was due to a lack of time spent on the explanation by the nurse-midwife. One woman described this as, *“Sometimes you ask the nurse-midwives and sometimes they don’t give you much attention because they have a lot of patients. Sometimes they don’t have a moment to tell you, to help you understand, and sometimes you leave with doubts,”* (Participant 7, LTFU). In other cases, the lack of understanding was due to forgetting much of the nurse-midwife’s explanation. One woman stated, *“Yes, they explained [the HPV test] to me, but I forgot,”* (Participant 9, LTFU) while another stated: *“To be honest with you, I don’t remember it so well, but I was told that it was to rule out some diseases like cancer or venereal diseases,”* (Participant 3, LTFU).

Seven women were anxious or scared about their result or specifically feared cancer. One woman described her fear, *“I felt bad, and I was afraid, and I knew I was going to have cancer. It was very hard … The first thing that came to my mind was to think that I was going to die,”* (Participant 13, completed treatment).

Another woman described how her friends told her that if she went for treatment, she would find out she has cancer:

“Don’t go, you will really get cancer. They are going to put an ugly thing in you, like this. They are going to take out your uterus, oh, no, no, no, don’t go”. Yeah, I also cowardly said, “I’m not going to go.” I was afraid.

(Participant 11, LTFU)

During a discussion of the process to receive HPV results, six women mentioned confusion about how to receive results. In some cases, women stated that they expected a house visit or phone call to receive their results and did not get one: *“Because the lady told me that if I have something, she will come and look for me. But I, well, I said to myself that I didn’t have anything. Why? Because she didn’t come looking for me,”* (Participant 9, LTFU). In other cases, women were unsure how to receive their results:

*At the health post, when I did it [the HPV test], they didn’t tell me to come back, and I thought that they would tell me something … because the lady didn’t tell me, “You are going to come on such and such a day to find out about your test.”* (Participant 20, LTFU)

When discussing treatment, 10 women showed a lack of understanding of treatment and its possible side effects. One spoke about concerns of sterilization with treatment: *“That has been my doubt and when they say ‘sterilization’, ‘cauterization’ and all that”* (Participant 4, LTFU). Two of these women expressed confusion about whether a treatment was available, with one woman asking the interviewer: *“I would like to ask you a question, does this disease have a cure?”* (Participant 7, LTFU).

Eight women discussed fear or anxiety about treatment. One woman stated, *“I am so afraid of the little machine [thermocoagulator],” (Participant 9, LTFU), while another stated, “I’m a little scared, I am. I’ve never done this, and it scares me a little bit,”* (Participant 23, LTFU).

#### Structural:

The main structural barrier was long wait times for receiving HPV results or follow-up care. Six women reported challenges with completing the continuum of care due to long wait times. Four of these women spoke about delays in receiving their HPV result. One woman stated, *“They told me to go to the health post, and when I went to ask, they told me that the results were not available,”* (Participant 20, LTFU), while another stated, *“I went twice to ask the lady if my result had arrived. She told me it hadn’t,”* (Participant 9, LTFU).

#### Other:

The main other barrier was more urgent work matters, with five women mentioning a lack of time due to more urgent work. One woman described her priority of work as: *“I never went, because of work I have not gone* (Participant 1, LTFU). Another stated: *“I work, Miss. I sell. At the end of the day, I sell. I go to sell on the street. That’s why I haven’t gone,”* (Participant 15, LTFU).

#### Financial:

A minority of women (two of the 24 women) mentioned financial barriers. One woman spoke about not having money to travel to the health center, *“I didn’t have the money to go. That’s why I haven’t gone”* (Participant 12, LTFU). Another spoke about the opportunity cost as a result of missing work: *“If I don’t sell, my children don’t eat. If I don’t wash other people’s clothes, they don’t eat either, so how could I go?* (Participant 11, LTFU).

#### Facilitators of follow-up care:

Despite the barriers to follow-up care discussed above, women interviewed discussed two main facilitators to completing the continuum of care: good knowledge of or a desire to better understand HPV and its treatment.

Eight women showed a good understanding of HPV and its treatment, often due to a good explanation from the nurse-midwives. One woman demonstrated her understanding of HPV: *“He told us that this requires a treatment because if we don’t have a treatment, it can advance. If you don’t realize it, as cancer is silent, it can arrive even when you are in the last stage,”* (Participant 6, completed care). One woman described a helpful explanation from the nurse-midwife: *“She took a good look at my face, she told me that I do have the beginnings of cancer, “pre-cancer” she said, “no, the cancer is not there yet. You have pre-cancer. You still have time to get it fixed because you are young. You are strong,”* (Participant 11, LTFU).

Additionally, five women showed a desire to learn more about HPV and its treatment. One woman asked the interviewer for more information about HPV: *“Can my partner also have that [HPV]?”* (Participant 3, LTFU). Another woman described looking for information on the internet: *“I went and checked on the Internet: what is it, why and how come, and all those things,”* (Participant 4, LTFU).

#### Natural medicine:

Ten women spoke about taking natural medicine as a supplement to the care provided in the public healthcare system. Seven of these women had not yet received treatment but stated that they would like to receive treatment during their interview. These women often reported taking natural medicine to address symptoms they were experiencing. One woman stated, “I took natural medicine for the pain,” (Participant 9, LTFU). Three of these women had already received treatment and took natural medicine to improve their post treatment healing: “That is why I continue with natural medicine and with my treatment,” (Participant 22, completed care).

#### Nurse-midwives’ and women’s outcomes:

**Figure 7:**
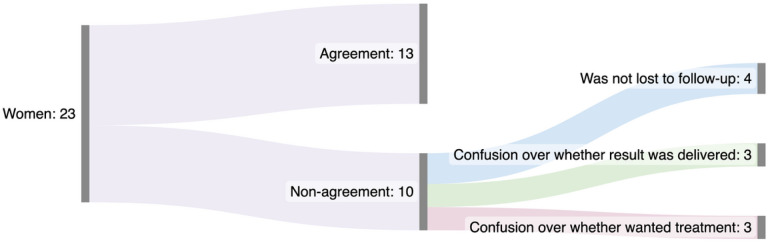
Depiction of the agreement and disagreement in reasons for loss to follow-up between nurse-midwives and women

When comparing data from nurse-midwives’ interviews with women’s interviews, we found agreement in the reason why women were LTFU in 13 cases, non-agreement in 10 cases, and encountered missing data from the nurse-midwife interview in one case. Out of the 10 cases of non-agreement, four women had received treatment according to the women, but the nurse-midwives did not have documentation of this; three women stated that they had not received their positive HPV results, despite the nurse-midwife stating that they had received it; three of the women stated that they wanted to receive treatment, while the nurse-midwives stated that they did not want to receive treatment (Fig. 7).

## Discussion

An important finding in this study was the impact of the absence of a complete registry for managing appropriate follow-up care for HPV positive women. Despite efforts to develop and utilize a registry system, as well as manual searches for data at healthcare facilities, there were no records of women in the study completing care prior to the interviews. The nurse-midwives, who coordinate much of the follow-up care, also often had incomplete or inaccurate data on women’s follow-up, including instances where they had no registration of women’s HPV results and instances of mistakenly recording women as having received results when they had not. The fact that some women complete their care in private settings makes registration of follow-up even more complicated. Additionally, databases for monitoring screening and treatment data were fragmented between primary and hospital-level care, making it challenging to determine if patients referred to the hospital received follow-up care, including women in our study who received undocumented hospital care. While this fragmentation has been seen previously in the MRIS and in other LMICs (10, 24), this study also revealed instances where registration of treatment was missing at the primary-level. Successful EDT programs need data registries that are consistently used by all relevant health professionals at the primary- and hospital-levels with accurate documentation of follow-up care linked across levels of care. Implementation science frameworks can be used, including Participatory Action Research, to improve use of registry systems by allowing stakeholders to internally derive registry systems and feel ownership over the new system (25, 26).

Women who were LTFU expressed a desire for treatment but faced various barriers throughout the continuum of care, starting with receiving their results. Nurse-midwives and our team experienced challenges in contacting these women due to invalid phone numbers or an inability to locate them at their registered address. Conducting house visits was time-consuming and further complicated by the possibility of women being away during the visit. We also observed instances where nurse-midwives and our team successfully located women that the other could not, indicating transient dynamics within these communities where women are not consistently found. Relatedly, some women assumed that if they were not visited by a nurse-midwife, everything was fine, while others did not know when or how to pick up their results. For women who went in person to pick up their results, some women described long wait times. Far too often, these factors culminate in women being unable to receive their results in a timely manner or altogether. Long wait times and challenges in delivering results are barriers seen in LMICs (11, 24, 27). However, the result that only a minority of women experienced long wait times is a positive shift from our previous research in the MRIS which found that half of women (10 out of 20) experienced long wait times (10). The challenge of timely results delivery or delivery of results at all can be addressed through greater emphasis on information collection from women, including accurately recording full addresses or asking women to provide a second phone number (e.g., a landline). Alternatively, at the time of screening, women could be provided with a phone number to call to receive their results and speak to a trained professional. This person could be a ‘patient navigator’, who can help guide women through the follow-up care process (28). Patient navigators have been shown to increase care completion rates following positive cancer screenings (29, 30). Importantly, the patient navigators do not need to be clinical staff but instead can be trained to coordinate care, provide health education and information, and offer counseling and psychosocial support (29).

The women and nurse-midwives also outlined cognitive barriers to completing the continuum of care including a lack of understanding and fear or anxiety about HPV results and treatment. In some cases, cognitive barriers arose due to nurse-midwives being too busy to provide detailed counseling. In other cases, women forgot information shared during counseling. Importantly, during implementation of the screen-and-treat program in the MRIS, Proyecto Precancer provided counseling training to nurse-midwives that aimed to address many of these cognitive barriers, which were previously identified in the MRIS and other LMICs (10, 11, 14, 15, 20). While this counseling training may have addressed some cognitive barriers – as seen by women in this study who discussed facilitators for care (e.g., a good understanding of HPV) – this study highlights the importance of further improving counseling following HPV testing, including addressing nurse-midwives’ time constraints, reducing fear and anxiety, and addressing women forgetting information. Patient navigators could be trained to provide counseling that specifically addresses fear and anxiety around HPV, alleviating the time constraints faced by nurse-midwives. Guidelines and tools can also be developed for patient navigators to promote consistency in key messages and reduce the risk of confusion (31). The tools can include take-home health education materials, which can be adapted to the local and cultural context and provide information on HPV, its treatment, and the process of seeking follow-up care. Traditional health education methods, such as take-home counseling materials, have been shown to improve health literacy in LMICs (32) and decrease anxiety and increase knowledge following abnormal cervical cancer screenings (33).

Financial barriers in this study were minimal; nurse-midwives reported that no women were LTFU due to financial barriers, while two (out of 24) women reported financial barriers. This is a substantial shift in barriers from our previous work in the MRIS at the hospital-level which found that 14 (out of 20) women faced financial barriers (10).

Women in this study also commonly mentioned a lack of time due to more urgent work matters as a barrier. This has been found in other LMICs (16, 27, 34), and previous research in Latin America suggests that informal workers have fewer social protections to allow them to leave work to attend follow-up cervical cancer preventative care (16). In Iquitos, much of the economy relies on informal work, and further research can explore support options for women unable to attend follow-up care due to work obligations.

The women in this study reported using natural medicine as a supplement to care in the healthcare system while nurse-midwives reported a minority of women used natural medicine instead of care in the healthcare system. In Peru, natural medicine has been found to be used in conjunction with care in the healthcare system or only when the barriers to receiving follow-up care were too great to overcome (10, 35). The discrepancy between nurse-midwives’ reports and previous literature may be due to cognitive barriers where women did not fully understand treatment availability or miscommunication where nurse-midwives did not understand that some women, who they reported did not want treatment, did want treatment. Improved counseling, including take-home materials, may help ensure that nurse-midwives provide consistent and complete information and that women are fully informed about treatment availability (32, 33).

### Limitations:

Some women in this study were not LTFU, despite our inclusion of women who were recorded as LTFU. We included these women in the study as they add valuable information about the challenges with the current registry systems. The findings of this study may not be generalizable to other regions; however, they provide information on limitations faced in resource-limited, primary-level screen-and-treat systems.

## Conclusion

While implementation of a primary-level screen-and-treat program with HPV testing and ablative therapy reduced the LTFU rate from 69.8–30.0% in the MRIS (9), task shifting cervical cancer care to the primary-level did not entirely eliminate LTFU. Instead, this shift reduced barriers seen in the previous system including women’s anticipation of challenges with seeking follow-up care, burdensome multi-step care processes, and out-of-pocket payments (10). A holistic, systems thinking approach that considers multiple stakeholders’ perspectives - from women to nurse-midwives - is necessary for countries to meet cervical cancer elimination goals. Inclusion of both perspectives in this study elucidated barriers that would have otherwise remained hidden and highlighted the need for successful EDT programs to have complete registry systems with patient-level data linked across levels of care, strong counseling materials which may incorporate patient navigators and take-home materials, and support structures to address work related time constraints.

## Data Availability

Data and materials are available on request to the corresponding author.

## Abbreviations

HPV: Human papillomavirus
EDT: Early detection and treatment
LTFU: Lost to follow-up
VIA: Visual inspection with acetic acid
MRIS: Micro-Red Iquitos Sur
SIS: *Seguro Integral de Salud* [Comprehensive Health Insurance]
HCAB: Health Care Access Barriers
